# Supplementary material for: A possible thermodynamic definition and equation of state for a model of political election cycles
Source: PLoS One. 2024 Mar 11;19(3):e0300113. doi: 10.1371/journal.pone.0300113 (PMC10927116; doi:10.1371/journal.pone.0300113)
Supplement: Attachment — Submitted filename: ResponeToReviewers.pdf [file pone.0300113.s002.pdf]

Response to Reviewer 1

(overall summary of changes): The authors are grateful for the comments and address each of the points.

- 1. "The novelty of the paper can be stated more clearly."
  - (reply to the reviewer) This has been addressed within in the Introduction where more language was added to emphasize the novelty of the approach.
- 2. "The abstract can be revised to show the objective of the paper in a more evident way. The distinctive parts of the paper can be stated so that the difference of this paper with previous ones can be shown."
  - (reply to the reviewer) The this has been addressed in the abstract and Introduction now.
- 3. "I would also like to recommend English editing. For example, the first sentence in the abstract can be shortened or edited to make it clearer in meaning."
  - (reply to the reviewer) This has been edited.
- 4. "The authors can check if all the figures have been cited appropriately in the text."
  - (reply to the reviewer) The figure references have now all been checked.

Response to Reviewer 2

(overall summary of changes): The authors are grateful for the comments and address each of the points.

- 1. "The concept of temperature is defined as dQ=dS/T so T=dS/dQ, where dQ stands for the transfered heat or change of Hamitlonian energy. Such reasoning is not included in presented manuscript. Therefore usage of term temperature is not fully justified.
  - (reply to the reviewer) The overall aim is to produce a dimensionality reduction from the multiple variables of the microstates to more descriptive variables in a macroscopic description. The volume and pressure are defined the system. As such the dimensionality reduction can be achieved through an equation of state connecting the volume and the pressure and mandating a variable equivalent to temperature. Since the correlation between the volume and the pressure is categorically defined via the political vs the non-political actions the temperature definition must be such that it complies with this behaviors. While there is no singular way to define the temperature, in order to adapt the model to resemble an equation of state of an ideal gas the temperature would have to be analogous to the product of the volume and the pressure. Given the additive properties of the pressure and the volume on possible solution to transform this addition to a multiplication was to use an exponential operator. Therefore by doing the above we achieve that the microscopic behavior is adequately correlated to the pressure and the volume. And the pressure and the volume conform to a behavior similar to an equation of state of an ideal gas. As such the temperature is defined. While we do understand that this is not the only possibly way to define a thermodynamic system upon a social system we believe that it is adequate as it is able to capture the phenomena produced in the simulation as well as resemble standard thermodynamic equations. In this formulation of the model the energy is not defined and as a consequence the correlation of the temperature and

the energy is not a necessary. Considering the energy is part of future work and now mentioned in the final words of the text.

- 2. "In such a way Hamiltonian for used „CMI" model (C(x,y,t), M(x,y,t), I(x,y,t)) shall be identified. Then partition function denoted by Z shall be determined. Even Wikipedia gives broad description of this metodology. Equation of State is not fully justified. The manuscript cites: T=exp(n_total), P=exp(n_pheripheral), V=exp(n_political), PV=T. Therefore, manuscript implies operator equation: exp(n_pheripheral)exp(n_political)=exp(n_total). How such operator equation can be justfied. The Question is operator equation n_pheripheral+n_political=n_total. If commutator [n_pheripheral,n_political] is equal to zero?"

  - (reply to the reviewer) Although a very interesting idea to formulate the Hamiltonian, the scope is directed at only the thermodynamic perspective. The Hamiltonian could potentially allow more accurate representation of social dynamics among agents but for this work the thermodynamics is the main scope. As such the Z can be omitted without considering possible further representations. The equation of state is justified using the above response which is based on the aspect that the energy for this model is not defined. It is a possible avenue for future work to consider the energy formulation based on agent state but in this approach no such proposals are made. This allows the current equation of state to be valid given the variables included. The relationship n_pheripheral+n_political=n_total is contained in the model and both quantities can be known allowing for those statements to hold.

- 3. "Schroedinger equation indeed is operator equation: $Ekinetic = [p^2/(2m)], Epot = V(x), Etotal = Ekinetic+Epot$. So if commutator [Ekinetic,Epot]=0 we can write exp(Ekinetic)exp(Epot)=exp(Etotal) and nonzero commutator [Ekinetic,Epot] not equal 0, so exp(Ekinetic+Epot)=exp(Etotal) In Quantum Mechanics we have for example H(t)|psi(x,t)>=i*hbar (d/dt)|psi(x,t)> leading to |psi(x,t)>=exp[(1/i*hbar)Int(H(t1),t0,t)]|psi(x,t0)>. The title supposed to be changed into „A Possible Thermodynamic Definition and Equation of State for a Model of Political Election Cycles"."

  - (reply to the reviewer) In QM the kinetic and potential energy are independent in their definitions and as such their commutator is zero. Similarly in our case, the pressure and the volume can, by definition, be know simultaneously. Therefore, their commutator is zero and therefore the equation exp(P)exp(V) = exp(T) can be written. This also conforms to the simulation rules as well.

- 4. "The manuscript shall cite additional positions: P1) Van Kampen, „Stochastic Processes in Physics and Chemsistry". → proper definition of temperature

  P2) „Thermodynamics in Stochastic Conway's Game of Life" [https://www.mdpi.com/2410-3896/8/2/47]→ methodology of thermodynamical description of Game (as Election Process) is given with proper definition of temperature for given cellular automata system.

  P3) „Equivalence between finite state stochastic machine, non-dissipative and dissipative tight-binding and Schrödinger model"(doi: 10.1016/j.matcom.2023.02.018) → methodology of mapping"

  - (reply to the reviewer) These highly relevant papers have now been included in the text emphasizing their relevance and merit.

References
