# Supplementary material for: A possible thermodynamic definition and equation of state for a model of political election cycles
Source: PLoS One. 2024 Mar 11;19(3):e0300113. doi: 10.1371/journal.pone.0300113 (PMC10927116; doi:10.1371/journal.pone.0300113)
Supplement: Attachment — Submitted filename: Response to Reviewers2.pdf [file pone.0300113.s003.pdf]

Response to Reviewer 2

(overall summary of changes): The authors are grateful for the comments and address the point raised.

- 1. Due to this fact I recommend to add one sentence in conclusion section: "Further justification of usage of thermodynamic cycles methodology in political election cycle shall be attempted with introduction of definition of effective Hamiltonian (that is dependent on (C,M,I)), so Hamiltonian change dE accompanied with entropy change dS will lead to temperature dependence expressed by formula T=dE/dS ".
  - (reply to the reviewer) The text is now included in the Conclusion section.
